# Prophage diversity in poultry-associated *Salmonella enterica* from Ecuador: a case study using an *in-silico* terminase-based approach

**DOI:** 10.3389/fmicb.2026.1703134

**Published:** 2026-03-12

**Authors:** Francisco Quelal-Madrid, Carolina E. Armijos, Christian Vinueza-Burgos, Lorena Mejía, Sonia Zapata-Mena

**Affiliations:** 1 Instituto de Microbiología, Colegio de Ciencias Biológicas y Ambientales, Universidad San Francisco de Quito (USFQ), Quito, Ecuador; 2 Department of Biology, University of North Carolina at Chapel Hill, Chapel Hill, NC, United States; 3 Unidad de Investigación de Enfermedades Transmitidas por Alimentos y Resistencia a los Antimicrobianos (UNIETAR), Facultad de Medicina Veterinaria y Zootecnia, Universidad Central del Ecuador, Quito, Ecuador

**Keywords:** *Salmonella enterica*, serovar Infantis, genomes, *in-silico*, prophages

## Abstract

Prophages can constitute up to 30% of the accessory genome in *Salmonella* enterica, acting as major drivers of virulence evolution and antimicrobial resistance; however, their diversity and functional contribution in Ecuadorian poultry-associated lineages remain unexplored. Here, we analyzed 142 S. *enterica* genomes from poultry and clinical sources to systematically characterize prophage diversity and cargo gene content. Genomes were assembled using SPAdes and screened with Phigaro and PHASTEST, while virulence-associated genes were identified through VFDB and VirulenceFinder. We identified a high prevalence of *Peduovirus* pro483 in S. *Infantis* isolates, carrying cargo proteins such as metalloendopeptidase, whereas related S. *Enteritidis* strains harbored distinct cargo elements, including cytosine-specific methyltransferases, consistent with independent horizontal acquisition events. Notably, *Enterobacteria* phage ST104 was detected in S. *Typhimurium* isolates encoding superinfection exclusion proteins (SieA and SieB), suggesting enhanced resistance to secondary phage infection and potential competitive advantages within microbial communities. Collectively, these findings provide the first comprehensive characterization of prophage diversity in S. enterica from Ecuadorian poultry production systems and underscore the role of prophages as dynamic contributors to lineage-specific adaptation, virulence potential, and public health risk.

## 1 Introduction

Phageomes are estimated to comprise approximately 80% of the virome in some environments, particularly gut microbiomes (Dion et al., 2020; Henrot and Petit, 2022; Wendling, 2023) and can represent >20% of their host’s genome (Dion et al., 2020; Nishijima et al., 2022; Wendling, 2023). Prophages are specific forms of bacteriophages that are integrated into the bacterial genome as lysogens and remain latent until certain environmental or host physiological conditions trigger their excision (Dion et al., 2020). While residing latent in a genome, prophages can influence several aspects of their host’s physiology, such as virulence, metabolic intake, antimicrobial resistance, and phage infections, making them an interesting focus, especially for gastrointestinal tract pathogens like *Salmonella* (Wahl et al., 2019; Tisza and Buck, 2021).

In *S. enterica*, prophages constitute up to 30% of the accessory genome and are recognized as one of the main contributors to the species’ diversity (Wahl et al., 2019). Their impact has been traditionally studied through the expression of virulence genes and other characteristics that affect the adaptation of *S. enterica* to different environments, but recent studies have focused on the influence of prophages in the infection dynamics, through the expression of genes that confers resistance to certain groups of phages (Wahl et al., 2019; Dion et al., 2020; Henrot and Petit, 2022). A clear example is observed in many p22-like phages which harbor superinfection exclusion genes that prevent the infection of multiple phages by inducing abortive infection or surface antigen modification (Owen et al., 2021). Research on major serovars such as Enteritidis, Typhimurium, and Infantis has primarily focused on prophages as evolutionary markers, given their tendency to become fixed in populations. However, studies examining the specific role of prophages in shaping serovar adaptation remain scarce. It has also been observed that prophage φSE12 in *S. enteritidis* confers specific virulence to mice, specifically through the expression of *sodCI* and *sopE* genes, which also have been observed in other prophages like *Gifsy-2* in *S. Typhimurium* conferring macrophage invasion properties and subsequent systemic infections in mice and other animal models (Araya et al., 2010). In *S. infantis*, data regarding prophages is still scarce but there are studies linking the presence of pESI-like plasmids to specific prophage lineages like P2-like phages, helping clarify the adaptation mechanisms and the polyphyletic nature of this serovar (Gymoese et al., 2019; Andrews et al., 2024).

Understanding the influence of prophages in *S. enterica* is crucial for comprehending the emergence of certain strains or serovars. This influence operates through the expression of virulence genes, promoting the success of these strains in specific environments (Weissman et al., 2018; Gymoese et al., 2019; Cohen et al., 2020; De Sousa et al., 2021). However, despite their recognized impact, the presence, distribution, and functional roles of prophages in *S. enterica* genomes remain largely unexplored. The limited number of studies addressing this aspect highlights significant knowledge gaps, particularly regarding the variability of prophage content across different serovars and its potential contribution to bacterial adaptation and pathogenicity.

Central to this contribution to pathogenicity are the cargo genes carried by prophages—accessory elements like antimicrobial resistance and virulence factors that provide significant fitness advantages to the bacterial host (Wahl et al., 2019). In *Salmonella*, acquiring these elements via horizontal gene transfer is a primary driver of ecological adaptation, allowing specific lineages to thrive in high-pressure environments (Wahl et al., 2019). Metagenomic data further shows these phages often carry metabolic enzymes that reshape host physiology to improve stress tolerance (Benler et al., 2021; Dion et al., 2020).

Identifying these elements relies on conserved structural markers. Terminase subunits are essential for DNA packaging and serve as hallmark genes that reflect the evolutionary history and taxonomy of the phage (Benler et al., 2021; Evseev et al., 2024; Wangchuk et al., 2021). Complementary to this, integrases facilitate site-specific recombination and act as robust molecular markers for identifying temperate phages (Balding et al., 2005). In *S. enterica*, integrase typing is a particularly useful indicator of overall genomic diversity (Colavecchio et al., 2017). Integrating these conserved markers with variable cargo features allows for a comprehensive understanding of prophage-driven diversity.

This study implemented specialized bioinformatic tools—such as Phigaro, a customized *S. enterica* prophage database, BLAST, and a terminase-based approach—to characterize the prophage landscape across 142 *S. enterica* genomes from a high-priority Ecuadorian cohort (Mejía et al., 2020). By focusing on this specific population from poultry farms, carcasses, and clinical cases, we aimed to validate our methodology for identifying prophage-encoded virulence and diversity within a dominant regional lineage.

## 2 Materials and methods

### 2.1 Genome assembly

**Figure 1:**
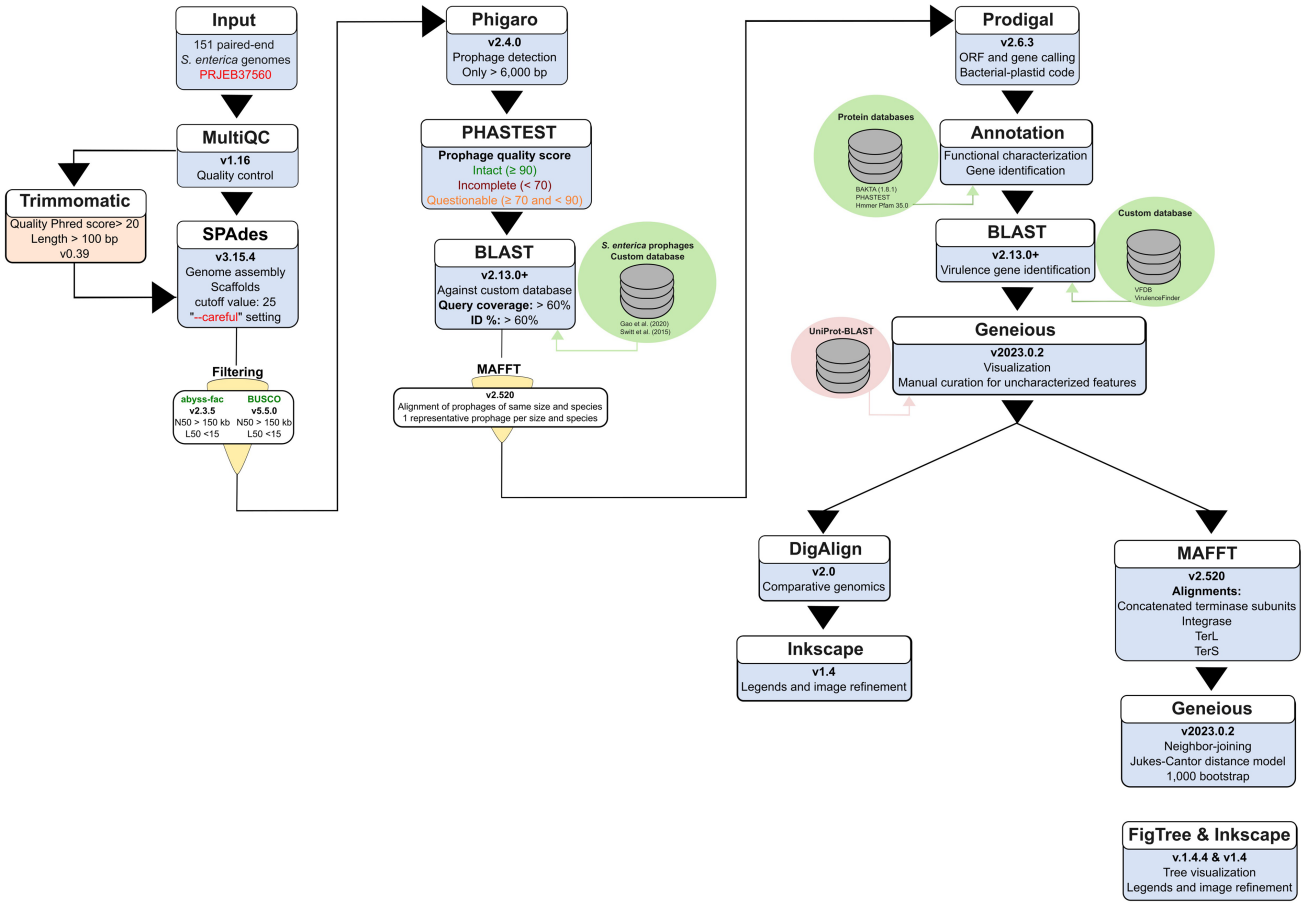
Flowchart depicting the bioinformatic pipeline used for the prophages detection, identification, annotation, classification, and taxonomic analysis performed in the *S. enterica* genomes from PRJEB37560. The flowchart details the software, tools, and databases employed. The order is indicated by triangles and arrows indicating the workflow.

The genome assembly protocol utilized 151 Illumina NextSeq paired-end sequences obtained from a previous study (Mejía et al., 2020), available under bioproject PRJEB37560 (Figure 1). Details regarding sequence names and serovars are provided in Supplementary Table 1.

Trimmomatic (v0.39) (Bolger et al., 2014) was employed to trim ambiguous nucleotides from paired-end sequences, ensuring a Phred quality score >20 and a minimum length of 100 bp. Quality control was performed using MultiQC (v1.16) (Ewels et al., 2016), and assembly was performed using SPAdes (v3.15.4) (Prjibelski et al., 2020), employing a cut-off value of 25 and the “--careful” setting to reduce mismatches and short indels. Only assembled scaffolds were utilized to prioritize the identification of large, non-fragmented prophage genomes (Figure 1).

Assembly quality was assessed with the abyss-fac utility of ABySS (v2.3.5) (Jackman et al., 2017) and BUSCO (v5.5.0) (Manni et al., 2021). To ensure prophages were detected within highly contiguous bacterial frameworks and to minimize the risk of recovering partial or fragmented viral sequences, we selected genomes meeting the following stringency criteria: <150 scaffolds, N50 > 150 kb, L50 < 15 (representing the number of scaffolds required to constitute 50% of the genome), genome completeness >85%, and a genome size >4.1 MB (Figure 1; Supplementary Table 1).

### 2.2 Prophage sequences identification

Assembled genomes were analyzed for prophage sequences, which were identified using Phigaro (v2.4.0) (Starikova et al., 2020). Prophage sequences shorter than 6,000 bp were excluded, as the shortest entry in our database was 6,408 bp. All prophage sequences obtained with both tools^1^ were concatenated into a single file to assess their completeness and quality using the PHASTEST online tool (Arndt et al., 2019). Briefly, prophage sequences uploaded to PHASTEST are classified based on a score: “intact” (score ≥90), “questionable” (score ≥70 and <90), and “incomplete” (score <70). This score is determined by the identity percentage of identified phage proteins, the number of phage features found in the query sequence, and the size of the prophage sequence (Arndt et al., 2019) (Figure 1).

Identified prophage sequences were extracted from the *Salmonella* genomes for prophage species identification using a custom database with BLAST command line applications (v2.13.0+) (Altschul et al., 1990; Morgulis et al., 2008). The foundational dataset for prophage identification was constructed by merging 160 reference prophage genomes retrieved from prior studies by Switt et al. (2015) and Gao et al. (2020) (Figure 1). These sources established that the selected sequences were either exclusive to or primarily related to *S. enterica* (Supplementary Table 2). To ensure the current accuracy of this database, we manually verified the completeness and species assignment of each reference genome by cross-referencing them against the NCBI Nucleotide and RefSeq databases (Sayers et al., 2022) and the most recent International Committee on Taxonomy of Viruses (ICTV) taxonomy. Species identification was performed using blastn with default parameters. While previous studies have utilized match thresholds of 45–58% query coverage with >90% identity, we adopted a more stringent threshold of >60% for both identity and query coverage (Figure 1). This decision was made to ensure higher confidence in our subsequent comparative analyses and to account for the structural polymorphism observed in our isolates. Prophage sequences that were not identified using this curated custom database were further analyzed using the NCBI virus database online BLAST service (Hatcher et al., 2017).

### 2.3 Prophage sequences annotation

To reduce redundancy while preserving structural diversity, identified prophage sequences were grouped by species and genomic length. Sequences within each group were aligned with their respective reference using MAFFT (v7.520) (Katoh et al., 2019) to verify identity. Subsequently, a single representative prophage was selected from each unique size-variant group for comprehensive genome annotation. This approach allowed us to account for significant structural polymorphisms—most notably in *Peduovirus pro483*—while maintaining a computationally efficient and non-redundant dataset for comparative analysis. Representative prophage sequences had their genomes annotated using Prodigal (v2.6.3) (Hyatt et al., 2010) for gene and ORF calling, specifying a bacterial-plastid genetic code. To provide a high-confidence functional characterization, gene sequence identification was performed using BAKTA (v1.8.1) (Schwengers et al., 2021), PHASTEST (Arndt et al., 2019), and the HmmerWeb Pfam 35.0 database (Potter et al., 2018; Figure 1).

Detection of virulence genes in our prophage sequences was accomplished by creating a custom database (see Footnote 1). This database was built using the Virulence Factor Database (VFDB) (Liu et al., 2022) and the VirulenceFinder database from the Center of Genomic Epidemiology at the Technical University of Denmark (DTU) (Malberg Tetzschner et al., 2020) and was built using BLAST command line applications (v2.13.0+) (Altschul et al., 1990; Morgulis et al., 2008), as previously described (Figure 1).

### 2.4 Prophage comparative genomics

Annotated genomes were visualized and curated using Geneious Prime (v2023.0.2) (Kearse et al., 2012). During manual curation, conflicts in gene naming or function were resolved by prioritizing annotations with the highest bit-score and lowest E-value across the three platforms. Hypothetical gene sequences were aligned against the UniProt (Coudert et al., 2023) database using the BLAST online service to minimize uncharacterized features. Curated annotated genomes were compared with their respective reference using DigAlign (v2.0) (Nishimura et al., 2024) to detect recombination and moron elements. Comparison graphics were refined using Inkscape (v.1.4) for clarity (Figure 1).

### 2.5 Prophage classification and taxonomy analysis

Prophage sequences were analyzed to determine their relationships with other related prophage groups and to corroborate their taxonomic assignments. Representative prophages previously obtained from the comparative genomics sequences were grouped with other taxonomically related phages from the ICTV to assess their relationships within their corresponding prophage group (Supplementary Table 2). Subsequently, the amino acid sequences for the terminase subunits (large and small) and the integrase were extracted from each prophage group. Following the methodology proposed by Wangchuk et al. (2021), the terminase subunits were utilized due to their conserved functionality across the primary bacteriophage groups, providing a reliable marker for phylogenetic reconstruction. Additionally, the integrase was utilized as a marker to assess prophage diversity, as established by Colavecchio et al. (2017).

The extracted sequences were aligned using MAFFT (v7.520) (Katoh et al., 2019). These alignments (see Footnote 1) were used to construct Neighbor-Joining trees using the Jukes-Cantor genetic distance model within Geneious Prime (v2023.0.2) (Kearse et al., 2012). To evaluate the reliability of the branching patterns, 1,000 bootstrap replicates were performed for each tree. Four phylogenetic trees were constructed: one using concatenated amino acid sequences of both terminase subunits (TerL and TerS), one using integrase (Int) sequences, and individual trees for the Terminase large subunit (TerL) and Terminase small subunit (TerS). The resulting phylogenies were visualized and curated using FigTree (v1.4.4) (Figure 1).

## 3 Results

### 3.1 *Salmonella* genomes and prophage identification

After removing duplicated or ambiguous reads from a total of 151 *S. enterica* data sets, the remaining reads were assembled into genomes. However, only 142 genomes met the required criteria for identifying unfragmented prophage genomes. These criteria included having < 150 scaffolds, an N50 > 150 kB, an L50 < 15, genome completeness > 85%, and a genome size exceeding 4.1 Mb. (Supplementary Table 1; Supplementary Figure 1).

The 142 assembled genomes exhibited an average of 152.63 scaffolds, with an N50 of 153,849 bp and an L50 of 6.62. The assembled genomes maintained an average genome size of 4.71 Mbp (Supplementary Figure 1). Among these, 136 genomes were identified as *S. infantis* (131 from poultry farms and 4 from clinical isolates), 3 as *S. enteritidis* (1 from a poultry farm and 2 from clinical isolates), and 3 as *S. Typhimurium* (all from poultry farms) (Supplementary Table 2).

**Figure 2:**
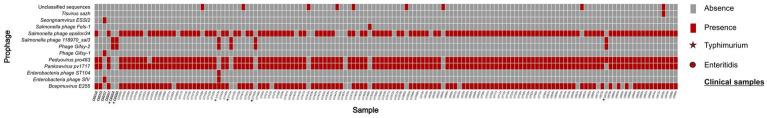
Heatmap representing the 12 distinct *Salmonella* prophage-associated sequences identified across the 142 *S. enterica* genomes. Red indicates the presence of a prophage sequence, while gray indicates its absence. Specific symbols indicate clinical samples from *S. Typhimurium* (star), *S. enteritidis* (circle), clinical samples are underlined and in bold letters.

Twelve distinct *Salmonella* prophage-associated species were identified, with sequence identities ranging from 69.10 to 99.91% and query coverage from 12 to 100%. Across the 142 genomes analyzed, the distribution of prophage content varied: 45 genomes (representing the highest carriage rate) contained five prophage sequences, 71 genomes carried four, and 23 genomes harbored three. Notably, only one *S. infantis* genome (U1467s) did not harbor any identifiable prophage, while two other *S. infantis* isolates, U1412s and U1496s, carried only one and two prophages, respectively (Figure 2; Supplementary Table 3). The 135 *S. infantis* genomes harbored the highest number of unique prophage species identified in this study, with a total of 9 different prophage-associated sequences. The most prevalent prophage sequences were *Bcepmuvirus E255, Pankowvirus pv1717, Peduovirus pro483*, and *Salmonella phage epsilon34*. Each of these sequences presented different sizes within the genomes ranging from 45,903 bp to only 4,198 bp. While all detected fragments are documented for transparency (Supplementary Table 3), sequences below 6,000 bp were excluded from downstream functional and phylogenetic analyses in accordance with our quality control criteria. Additional prophage sequences were identified; however, these were present in only a single genome, like *Phage Gifsy-1, Salmonella phage Fels-1*, *Seongnamvirus ESSI2*, and *Tlsvirus sazh* (Table 1).

**Table 1:** Distribution of prophage sequences detected in each *S. infantis* genome.

Prophage	Reference size	Phigaro	*Salmonella* genomes		BLAST				PHASTEST
		Detected size	Farms *n* = 131	Clinical *n* = 4	Percentage ID	Query coverage	Max score	Total score	Quality
*Bcepmuvirus E255* (NC_009237)	37,446 bp	17,388 bp	15	1	69.1%	21%	732	1467	Intact
		20,441 bp	98	2	69.1%	18%	732	1467	Intact
*Enterobacteria phage SfV* (NC_003444)	37,074 bp	5,826 bp	0	1	76.29%	12%	2,143	744	Intact
*Pankowvirus pv1717* (NC_011357)	62,147 bp	23,765 bp	120	3	71.03%	43%	2,269	4443	Intact
		41,942 bp	1	0	85.94%	36%	2,269	10,128	Intact
		42,427 bp	6	0	85.94%	35%	2,269	9556	Intact
		43,182 bp	1	0	85.94%	34%	2,269	9556	Intact
*Peduovirus pro483* (NC_028943)	29,237 bp	6,603 bp	49	1	97.47%	99%	11,152	11,284	Incomplete
		20,172 bp *	47	1	97.82%	80%	21,830	41,710	Intact
		20,234 bp *	4	0	97.82%	81%	21,825	27,885	Intact
		31,653 bp *	76	2	97.82%	79%	21,830	41,710	Intact
*Phage Gifsy-1* (NC_010392)	48,491 bp	19,209 bp *	0	1	99.97%	100%	34,611	34,937	Intact
*Salmonella phage epsilon34* (NC_011976)	43,016 bp	17,887 bp *	115	3	94.18%	73%	18,444	19,774	Intact
*Salmonella phage Fels* (NC_027984)	42,723 bp	39,728 bp	1	0	90.16%	46%	16,992	23,754	Intact
*Seongnamvirus ESSI2* (NC_047854)	28,765 bp	30,206 bp *	0	1	75.03%	69%	8,342	15,129	Intact
*Tlsvirus sazh* (NC_048065)	49,665 bp	43,966 bp*	1	0	90.36%	87%	27,663	53,120	Intact

^*^Selected prophage lengths with an adequate identification percentage and query coverage for genome annotation and comparative genomics.

The remaining six *S. enterica* genomes included three *S. Typhimurium*, in which *Phage Gifsy-2* and *Salmonella phage 118970_sal3* were the most prevalent prophage sequences. Additionally, *Enterobacteria phage ST104* was identified as the only prophage with an integrity of 99.97% (Table 2). The other three genomes corresponded to *S. enteritidis*, which also carried *Phage Gifsy-2* and *Salmonella phage 118970_sal3*, while one *S. enteritidis* genome uniquely featured *Peduovirus pro483* (Table 3).

**Table 2:** Prophage sequences detected in *S. Typhimurium* genomes.

Prophage	Reference size	Phigaro	*Salmonella* genomes		BLAST				PHASTEST
		Detected size	Farms *n* = 1	Clinical *n* = 2	Percentage ID	Query coverage	Max score	Total score	Quality
*Bcepmuvirus E255* (NC_009237)	37,446 bp	16,145 bp	1	0	69.1%	21%	723	1401	Intact
*Enterobacteria phage SfV* (NC_003444)	37,074 bp	7,026 bp	1	0	76.29%	12%	424	744	Incomplete
*Enterobacteria phage ST104* (NC_005841)	41,391 bp	45,948 bp *	1	0	99.97%	82%	56,373	69,621	Complete
*Phage Gifsy-2* (NC_010393)	45,840 bp	26,850 bp	0	2	77.75%	44%	6,544	19,573	Incomplete
		30,910 bp *	1	0	99.91%	100%	55,641	56,114	Intact
*Salmonella phage 118970_sal3* (NC_031940)	77,375 bp	30,024 bp	1	0	99.85%	63%	45,786	88,322	Intact
		45,903 bp	0	2	96.23%	63%	21,618	79,948	Intact

^*^Selected prophage lengths with an adequate identification percentage and query coverage for genome annotation and comparative genomics.

**Table 3:** Prophage sequences detected in *S. enteritidis* genomes.

ID (NCBI reference code)	Reference size	Phigaro	*Salmonella* genomes		BLAST				PHASTEST
		Detected size (bp)	Farms	Clinical	Percentage ID	Query coverage	Max score	Total score	Quality
*Peduovirus pro483* (NC_028943)	29,237 bp	33,052 bp*	1	0	97.16%	73%	21,492	39,240	Intact
*Phage Gifsy-2* (NC_010393)	45,840 bp	16,403 bp	1	0	99.75%	58%	6,544	16,821	Intact
		26,850 bp	2	0	99.75%	44%	6,544	19,573	Intact
*Salmonella phage 118970_sal3* (NC_031940)	77,375 bp	37,156 bp	1	0	96.4%	63%	21,618	79,948	Intact
		44,678 bp	1	0	96.23%	63%	21,618	79,948	Intact
		45,903 bp	1	0	96.23%	63%	21,618	79,948	Intact

^*^Selected prophage lengths with an adequate identification percentage and query coverage for genome annotation and comparative genomics.

### 3.2 *Salmonella* prophages comparative genomics and gene identification

As shown in Tables 1–3, prophage sequences fulfilling the criteria of >95% identity and >65% query coverage were included in the analysis. A total of seven prophage sequences were compared with their respective reference genomes to identify cargo features (accessory genes non-essential for the viral life cycle that provide fitness advantages to the host as defined by Wahl et al. (2019) and Dion et al. (2020) and/or virulence genes).

**Figure 3:**
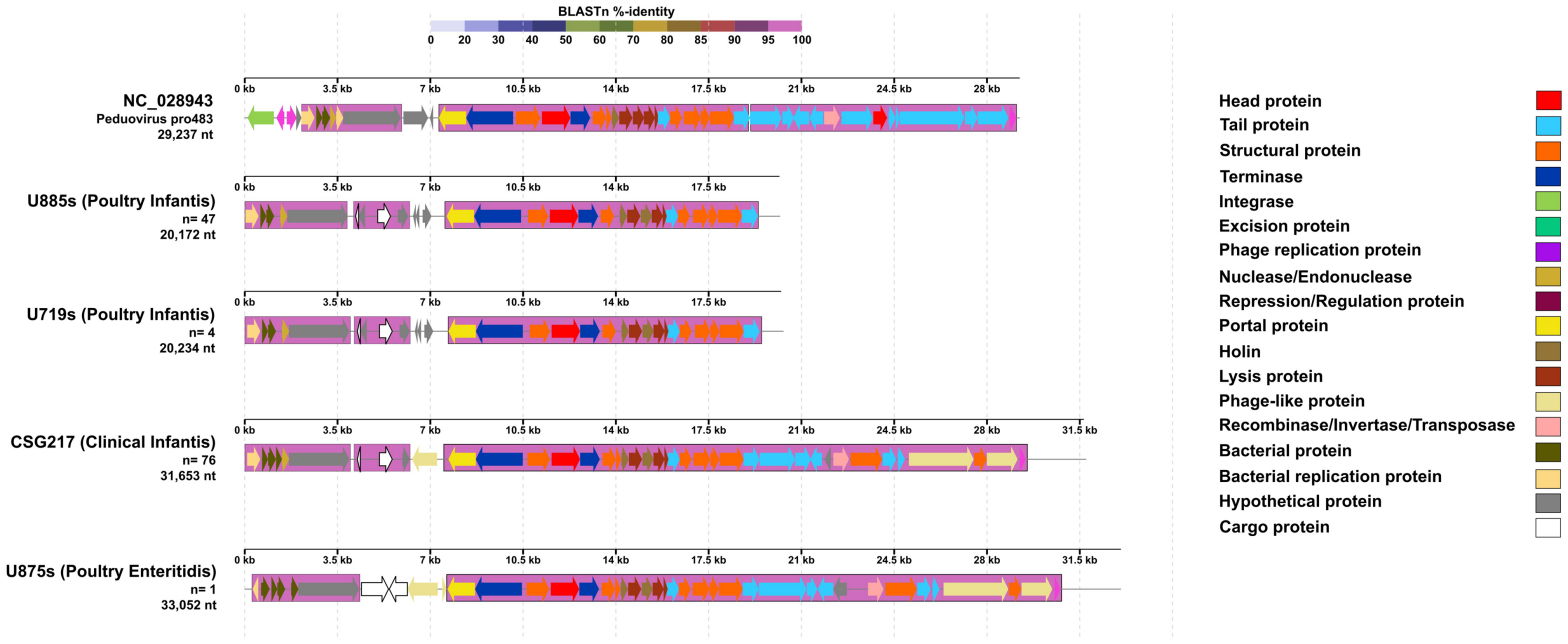
Comparative genomics graph of *Peduovirus pro483*-like sequences of varying sizes detected in *S. enterica*. Four representatives for each of the lengths found were selected, and their number of isolates harboring them is specified as “*n*.” The legend on the right indicates the main features identified for these prophage sequences compared to the *Peduovirus pro483* reference genome (NC_028943). At the top, a BLAST %-identity bar indicates the identity percentage of every genetic block or region compared to the reference genome, according to the color palette on the top. Obtained using DigAlign (v2.0).

The varying sizes associated with *Peduovirus pro483* were analyzed to identify cargo or virulence features —as previously defined— within the representative genomes (Figure 3). Two main genetic blocks were observed in common across all *Peduovirus pro483*-like sequences, exhibiting 100% identity to the reference Peduovirus pro483. In all *Peduovirus pro483*-like sequences found in *S. infantis*, regardless of size, two cargo proteins were consistently identified: an ImmA/IrrE-family metalloendopeptidase and a fimbrial protein (Table 4). Notably, the single Peduovirus pro483-like sequence identified in *S. enteritidis* shared conserved genetic blocks with those from *S. infantis* (Figure 3); however, it carried two distinct cargo proteins: a HEPN-MAE-28990 domain-containing protein and a cytosine-specific methyltransferase (Table 4).

**Table 4:** Cargo proteins and superinfection exclusion proteins detected in *Peduovirus pro483-like* prophage sequences.

Protein	Number of harboring genomes (Serovar)
Imma/Irre Metallo Endopeptidase	132 (*S. infantis*)
Fimbrial protein	
HEPN-MAE-28990domain-containing protein	1 (*S. enteritidis*)
Cytosine-specific methyltransferase	

**Figure 4:**
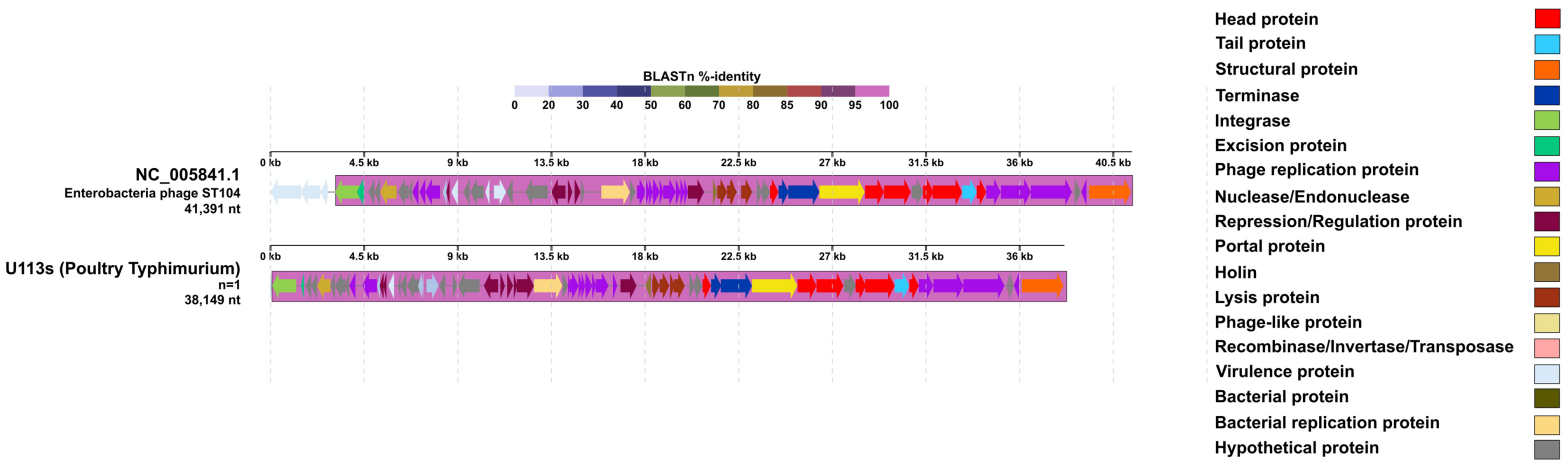
Comparative genomics graph of *Enterobacteria phage ST104-like* sequences of varying sizes detected in *S. enterica*. The legend on the right indicates the main features identified for these prophage sequences compared to the *Enterobacteria phage ST104* reference genome (NC_005841). At the top, a BLAST %-identity bar indicates the identity percentage of every genetic block or region compared to the reference genome, according to the color palette on the top. Obtained using DigAlign (v2.0).

**Figure 5:**
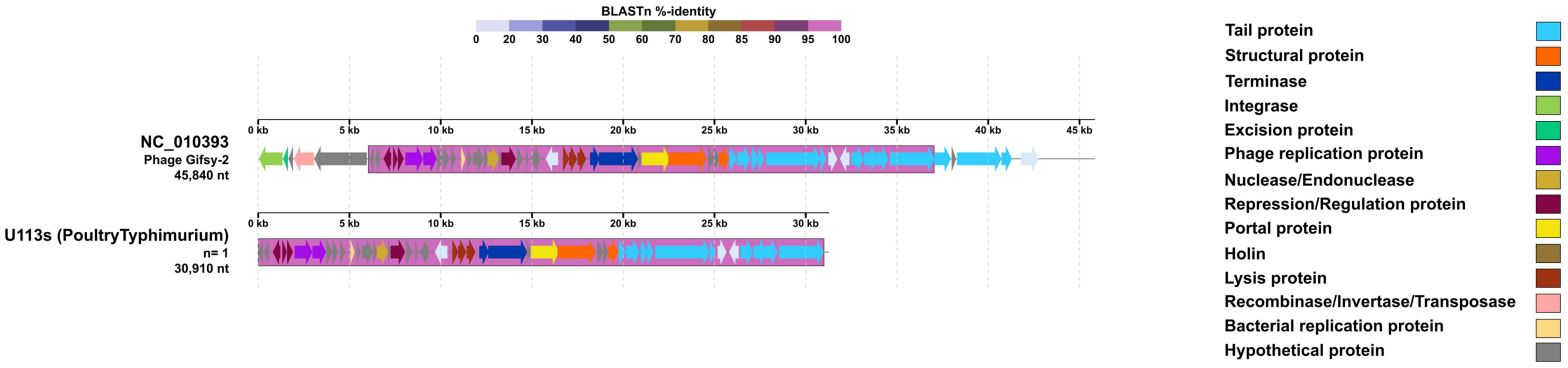
Comparative genomics of p*hage Gifsy-2*-like sequences of varying sizes detected in *S. enterica*. The legend on the right indicates the main features identified for these prophage sequences compared to the p*hage Gifsy-2* reference genome (NC_010393). At the top, a BLAST %-identity bar indicates the identity percentage of every genetic block or region compared to the reference genome, according to the color palette on the top. Obtained using DigAlign (v2.0).

The *Enterobacteria phage ST104-*like sequence from a *S. Typhimurium* showed a high level of identity and a significant number of conserved genetic blocks compared to its reference genome (Figure 4). Almost all the *Enterobacteria phage ST104* reference genome corresponded with a 100% identity, except for three genes corresponding to virulence proteins that were not present in our sequence. While features like excisionase, and some virulence factors were absent in our detected sequence, other features like SieA and SieB, both coding for superinfection exclusion proteins, and an integrase were present (Table 5). In the same sample, a *Phage Gifsy-2*-like sequence was identified, also with a high proportion of conserved genetic blocks (Figure 5) compared to its reference genome, and carrying 2 virulence factors: *SodC*, a superoxide dismutase, and GtgA, a type III secretion system protein (Table 5).

**Table 5:** Virulence proteins and superinfection exclusion proteins detected in prophage sequences in *S. Typhimurium* genome.

Protein	Number of harboring prophage
SieA	*Enterobacteria phage ST104-like*
SieB	
SodC	*Phage Gifsy 2-like*
GtgA	

Prophage-like sequences for *Salmonella phage epsilon34, Phage Gifsy-1, Seongnamvirus ESSI2*, and *Tlsvirus sazh* were also compared with their respective reference genomes, but no cargo, virulence factors, or superinfection exclusion proteins were detected. Relevant features as transposase were found in *Phage Gifsy-1* and *Tlsvirus sazh,* and an integrase in *Seongnamvirus ESSI2* (Supplementary Figure 2).

### 3.3 *Salmonella* prophages phylogenetic relationships

**Figure 6:**
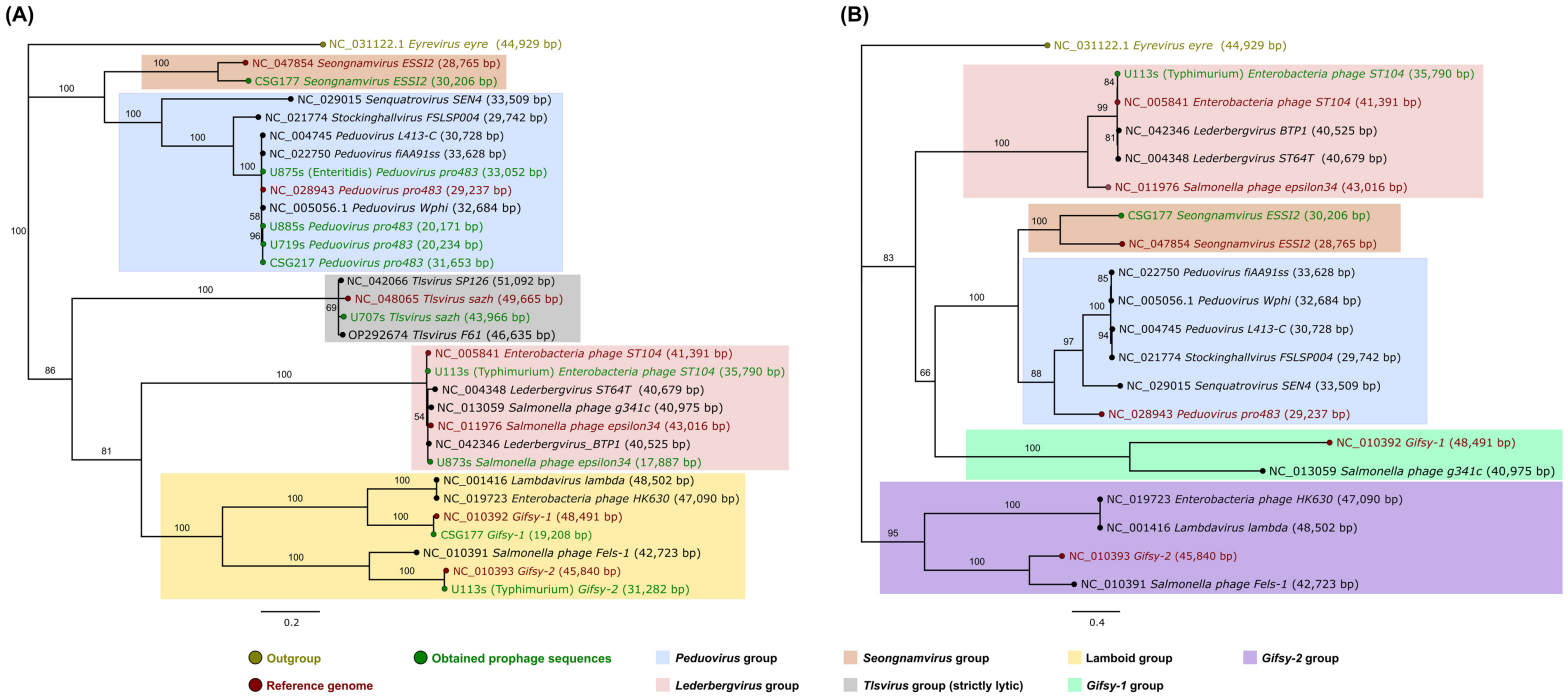
Neighbor-joining trees constructed with **(A)** terminase small and large subunits aminoacid sequences and **(B)** integrase aminoacid sequences. The horizontal branch lengths are proportional to the genetic distance (substitutions per site), and the scale bar for the branch length is indicated below each of the trees. Numbers at the internal nodes represent bootstrap support values derived from 1,000 replicates. Color-coded shading identifies major groups: *Peduovirus* (blue), *Lederbergvirus* (pink), *Seongnamvirus* (orange), *Tisvirus* (grey), *Lambdavirus* (yellow), *Gifsy-1* (turquoise), and *Gifsy-2* (purple). Outgroups are indicated in gold, reference genomes in red, and obtained prophage sequences in green. The concatenated sequences were extracted after annotation from every representative prophage sequence detected, their corresponding reference and other related prophage genomes. Analysis was performed in Geneious Prime (v2023.0.2) and visualized using FigTree (v1.4.4).

To determine the taxonomic relationships of the detected prophages, we performed a phylogenetic analysis using the concatenated amino acid sequences of both terminase subunits (TerS and TerL) and the integrase (Int) of prophage sequences positive for these features (Figure 6). The resulting phylogenetic trees provided key insights into the evolutionary placement of these prophages.

In general terms, the phylogenetic analysis based on the terminase subunits (Figure 6A) revealed five groups including the main prophages found. The first group encompassed our *Peduovirus pro483*-like sequences along with other *Peduovirus*-related representatives (depicted in blue in Figure 6A), as well as our *Seongnamvirus ESSI2*-like sequence and its corresponding reference (depicted in orange in Figure 6A). In a larger group related to the previous ones it was observed three subgroups: one featuring *Lederbergvirus*-related sequences (depicted in pink in Figure 6A), another containing lambdoid phages that included our *Phage Gifsy-1* and *Phage Gifsy-2*-like sequences (depicted in yellow in Figure 6A), and a third with *Tlsvirus*-related phages, including our *Tlsvirus sazh*-like sequence (depicted in gray in Figure 6A). Trees constructed with only the TerS (Supplementary Figure 3) and TerL (Supplementary Figure 4) sequences individually featured similar relationships as those previously described, but they showed lower support values.

Similarly, the analysis based on the integrase also yielded various groups (Figure 6B). However, in this tree, *Phage Gifsy-2*-related sequences formed a separate group from the rest of the representatives (depicted in purple in Figure 6B), although the internal relationships within the other clusters remained consistent with those observed in the terminase tree (Figure 6A).

Three *Peduovirus pro483*-like sequences were found to be closely related to *Peduovirus Wphi*; all of these originated from *S. infantis* (two from poultry farms: U885s, U719s, and one clinical isolate: CSG217). However, a *Peduovirus pro483*-like sequence detected in an *S. enteritidis* isolate (U875s) was not related to these three specific sequences but still grouped with the rest of *Peduovirus* representatives (Figure 6A). Although we did not detect integrase in our *Peduovirus pro483*-like sequences, a similar phylogenetic relationship was observed when using integrase as a marker, but only with the reference genomes: *Senquatrovirus SEN4* and *Stockinghallvirus FSLSP004* appeared more closely related to the other *Peduovirus* phages than *Peduovirus pro483*, yet they still clustered together (Figure 6B).

Our *Enterobacteria phage ST104*-like sequence was found to be identical to its reference. It clustered with other *Lederbergvirus*-related phages, including our *Salmonella phage epsilon34*-like sequence, though this grouping had a low support value (Figure 6A). A similar relationship was observed in the integrase tree (Figure 6B); however, in this instance, *Salmonella phage epsilon34* appeared less related to the *Lederbergvirus* group, despite still clustering with them.

## 4 Discussion

*Salmonella enterica* poses a significant public health concern due to its ability to adapt to diverse environments. This adaptability has been linked to the expression of virulence factors and other genetic traits. Recent studies have highlighted the role of prophages in modulating infection dynamics by encoding genes that confer resistance to certain phage groups (Dion et al., 2020; Henrot and Petit, 2022). In this study, we applied an *in-silico* framework to evaluate prophages within a selected cohort of *S. enterica* isolates from the poultry production chain in Ecuador. This targeted approach allowed us to resolve the prophage-host associations and virulence potential specific to this regional population, demonstrating the utility of our pipeline in a real-world surveillance context.

Prophages can significantly influence host fitness by encoding virulence factors that enhance colonization or genes that provide resistance to environmental stressors (Wahl et al., 2019; Dion et al., 2020). While these features offer potential evolutionary advantages, they may be lost or inactivated over time, a process often driven by the influence of other mobile genetic elements (Bobay et al., 2014; Gymoese et al., 2019). This evolutionary decay frequently results in the presence of defective or fragmented prophages (Wahl et al., 2019), as potentially observed in our *Peduovirus* pro483 and *Gifsy-2* analyses (Figures 3, 5), where sequences lacked essential integrase and excisionase proteins despite high identity to reference genomes. However, it is important to acknowledge that because these sequences were identified within assembled scaffolds rather than complete, closed genomes, some observed fragmentation may also be attributed to assembly limitations. Furthermore, the identification of a *Tlsvirus sazh*-like sequence—a *Drexlerviridae* phage typically considered lytic (Crossland et al., 2019)—carrying a transposase suggests the complex influence of mobile genetic elements in these regions (Supplementary Figure 2). Despite the significant fragmentation observed in the prophage genomes, our identification process revealed seven prophages with an identity and query coverage exceeding 60%. Among these, three—*Enterobacteria phage ST104, Peduovirus pro483*, and *Gifsy-2*—were particularly notable due to their high similarity to the reference genomes (approximately 99, 97, and 99%, respectively). These strong matches suggest a potentially greater functional relevance, particularly regarding their possible influence in *Salmonella*–host interactions (Dion et al., 2020; Henrot and Petit, 2022). The presence of *Gifsy-2*, for example, is consistent with previous studies linking this prophage to virulence traits in *Salmonella* (Wahl et al., 2019), lending support to the hypothesis that specific prophages may enhance the pathogenic potential of *S. infantis* in poultry-related environments.

Furthermore, these findings may suggest that selective pressures could be maintaining incomplete or defective prophages, contributing to the phage mosaicism, where gene blocks are conserved despite different evolutionary histories (Yu et al., 2017; De Jonge et al., 2019). This was observed in the various *Peduovirus pro483*-like sequences across our *S. infantis* genomes. Such phenomenona could potentially serve as markers for specific strains associated with particular environments (Bobay et al., 2014).

Among our *S. infantis* genomes, *Peduovirus pro483* was the most prevalent prophage sequence, suggesting a probable adaptation of this prophage to this serovar and its current environment. This is consistent with the significant role prophages play in *S. enterica* evolution, particularly in adaptation to specific serovars and environments (Switt et al., 2015; D’Alessandro et al., 2018; Wahl et al., 2019). Similar adaptation patterns have been reported for various members of the *Peduovirus* genus infecting *Escherichia coli,* especially those associated to clinical environments, and other pathogenic strains like *E. coli* O157: H7 or O145 (Pacífico et al., 2019; Shridhar et al., 2019). *S. infantis* have been recognized as the fourth most prevalent non-typhoidal serovar associated with human infections worldwide (Montoro-Dasi et al., 2023), with a prevalence of 83 to 98% in broilers and poultry farms from Ecuador and Peru (Vinueza-Burgos et al., 2019; Vallejos-Sánchez et al., 2019).

The high frequency of *Peduovirus pro483* in *S. infantis* may indicate that this prophage may has specifically adapted to this successful serovar within the poultry farm environment. *Peduovirus pro483* was originally isolated from an avian pathogenic *E. coli* and *S. Typhimurium* (Petrovska et al., 2016; Gymoese et al., 2019), it has also been reported clinical *E. coli* samples alongside P2-like and antimicrobial resistance genes (Rusconi et al., 2016; Pacífico et al., 2019;). Our phylogenetic trees, based on TerL and TerS amino acid sequences, confirmed that *Peduovirus* pro483 is indeed related to other P2-like phages, such as *Peduovirus* Wphi and *Peduovirus* fiAA91ss, which are associated with clinical environments (Pacífico et al., 2019; Kondo et al., 2021). These relationships could suggest a clinical origin for the acquisition of *Peduovirus* pro483 in *S. infantis*; however, additional data from clinical settings would be required to substantiate this hypothesis, as our current dataset included only six clinical *Salmonella* genomes.

Cohen et al. (2020) have reported *Peduovirus pro483* associated with *S. infantis* carrying pESI-like plasmids similarly to Mejía et al. (2020), but their analysis focused on genetic diversity and did not establish an association between the prophages and the pESI-like megaplasmids found. We delved deeper with our findings on *S. infantis* by providing data on an *S. enteritidis* genome also carrying *Peduovirus pro483*. While most of our samples were from poultry farms and chicken carcasses, 76 *S. infantis* (including the previously mentioned 3 clinical samples) and a poultry *S. enteritidis* presented a larger and more complete *Peduovirus pro483* genome. This could represent a novel adaptation of this prophage to *S. infantis*, given the similar origin of our genomes. This adaptation could be driven by two possible scenarios: selective pressures within the poultry farm environment, favoring prophage variants that enhance *S. infantis* fitness (Mottawea et al., 2018; Gao et al., 2020), or a recent acquisition of *Peduovirus pro483* given its integrity and maintenance of certain features (Bobay et al., 2014; Wahl et al., 2019; Henrot and Petit, 2022). Further studies are needed to identify the most likely scenario.

Peduovirus pro483 has been reported to carry the host epithelial cell invasion protein SopE, a hallmark of P2-like prophages identified in emerging clones within poultry environments (Dziva et al., 2013; Petrovska et al., 2016; Rusconi et al., 2016; Gymoese et al., 2019). However, SopE was not identified in any of the genomes analyzed in this study, neither within the *Peduovirus* pro483 sequences nor in other genomic regions. Instead, all *S. infantis* strains carrying this prophage harbored a fimbrial protein—a virulence factor that enhances surface adhesion and colonization (Huehn et al., 2010; Ksibi et al., 2022). Notably, Mejía et al. (2020) reported that the p-F219 megaplasmid in these genomes also carries fimbrial proteins, suggesting a reinforcement of colonization traits over invasion effectors.

Notably, the SopE effector was absent in both the Ecuadorian isolates and the *Peduovirus pro483* reference genome used in this study. This absence suggests that SopE may not be a core feature of this specific prophage lineage, or perhaps it was not harbored by the ancestral sequences at the time they were characterized and integrated into foundational databases. This reinforces the idea that the analyzed *S. infantis* populations do not represent the ‘emerging’ invasive clones often associated with SopE-mediated virulence. This pattern may represent a divergence from the prophage cargo typically reported in global epidemic lineages. Instead, the consistent presence of fimbrial cargo proteins indicates a localized adaptive strategy in the Ecuadorian poultry niche, where selective pressures favor environmental persistence and niche maintenance over initial host invasion (Bobay et al., 2014; Vinueza-Burgos et al., 2019). We acknowledge that the lack of SopE in the reference genome reflects a limitation of current databases, which may not yet capture the full range of prophage accessory variability across all regional lineages. Confirming this potential evolutionary trade-off will require further investigation using a more diverse dataset across different poultry-related serovars.

In our study, the *S. enteritidis* genome harboring Peduovirus pro483 lacked this fimbrial protein, hinting at serovar-specific variation. As *S. infantis* has become the dominant poultry serovar following *S. enteritidis* and *S. Typhimurium* vaccination (Montoro-Dasi et al., 2023), further data on recent population dynamics are essential. Such research will clarify how plasmid-prophage interplay contributes to the shifting epidemiological success or decline of these serovars.

The presence of *Enterobacteria phage* ST104 and *Phage Gifsy-2* in our *S. Typhimurium* isolates aligns with established reports of these prophages within this serovar (Ho and Slauch, 2001; Parker et al., 2021). While *Enterobacteria phage* ST104 has been classically associated with cattle and beef production environments (Parker et al., 2021), our identification of this prophage in poultry-derived isolates suggests that this scenario might have changed, indicating a potential broader ecological niche adaptation to the poultry environment. This is supported by the phylogenetic proximity of *Enterobacteria phage* ST104 to the *Lederbergvirus* genus, a group known for its high prevalence in poultry-associated serovars (Vaid et al., 2021).

However, it is important to note that this association is based on terminase-based phylogenetic analysis. While this indicates a clear taxonomic relationship through the sharing of conserved terminase subunits, comparing additional genomic features would be necessary to fully confirm this broader adaptation. Furthermore, although this prophage has been linked to antimicrobial resistance genes in other contexts (Colavecchio et al., 2017; Elayoni, 2021; Mohammed et al., 2023), no such resistance genes were detected in our sequences. Instead, we identified specific virulence factors associated with *Enterobacteria phage* ST104, consistent with the pathogenic potential typically described for members of the *Lederbergvirus* genus (Cohen et al., 2020). Studies in poultry environments with a more diverse range of *Salmonella* serovars and isolates would be required to confirm these hypotheses.

Conversely, *Phage Gifsy-2* has also been reported as a prevalent prophage in *S. enterica*, particularly within serovar Typhimurium (Ho and Slauch, 2001). Historically, *Phage Gifsy-2* is described as a lambda-like prophage with the potential to carry virulence factors and, in some cases, facilitate the mobilization or excision of other mobile genetic elements (Owen et al., 2020; Federici et al., 2021). The inclusion of virulence-associated cargo, such as those identified in our genomic analysis (Figure 5; Table 5), likely contributes to the selective pressure that maintains this prophage within *Salmonella* lineages (Foley et al., 2013). While our results confirm the presence of this prophage in the studied *S. Typhimurium* and *S. enteritidis* isolates, we acknowledge that our current analysis is focused on a specific population affecting the poultry sector. Because these serovars represent a smaller portion of our dataset compared to *S. infantis*, more extensive data from a broader range of sources would be required to establish a definitive conservation pattern. Future research incorporating induction assays would be necessary to determine if the specific sequences in our Ecuadorian isolates retain the capacity for excision or influence the integration of other prophages.

Prophage-encoded genes can influence *S. enterica* fitness by providing both immunity to phage superinfection and specific virulence factors. In our isolates, such genes were primarily identified within the genomes of *Enterobacteria phage ST104* and *Phage Gifsy-2*. Specifically, the *ST104* prophages featured superinfection exclusion genes, such as *sieA* and *sieB* (Figure 4; Table 5), which are recognized for their potential to protect the host from secondary infections by P22-like and lambdoid phages (Berngruber et al., 2010; Folimonova, 2012; Monteiro et al., 2019). Additionally, our *Gifsy-2* sequences harbored *gtgA* (a surface antigen modifier) and *sodC* (a superoxide dismutase), the latter of which may protect *S. enterica* from oxidative stress during intracellular infection (Ammendola et al., 2005; Golubeva and Slauch, 2006). Because this study focused on a specific population, these results may not fully capture the prophage diversity present across a broader range of poultry-related serovars. Furthermore, while these genes were identified genomically, functional gene expression assays are essential to confirm their active transcription and contribution to the fitness of these isolates.

Notably, while avian-related *S. Typhimurium* clones carrying *ST104* are frequently reported to also harbor the invasion protein SopE (Kirkwood et al., 2021), this virulence factor was absent in our *S. Typhimurium* dataset. This observation —consistent with the absence of SopE observed in our *S. infantis* isolates— combined with the presence of *sieA*, *sieB*, *gtgA*, and *sodC*, suggests a distinct functional profile for the prophages in our poultry-derived population. These findings are further supported by our phylogenetic analysis, where *ST104* clustered with *Lederbergvirus* BTP1, a phage known to utilize similar superinfection exclusion mechanisms to maintain host stability (Owen et al., 2021).

Our phylogenetic analysis provided support for the taxonomic associations of the prophages identified in this study. Based on the concatenated terminase subunits (Figure 6A), we identified the following groups: one encompassing all *Peduovirus*-related phages and *Seongnamvirus* ESSI2 (depicted in blue, and orange, respectively in Figure 6A), and another including *Tlsvirus*-related phages, lambdoid phages, and *Lederbergvirus*-related phages (depicted in gray, yellow, and pink, respectively in Figure 6A). In the first two groups, the association of *Peduovirus* with *Seongnamvirus* ESSI2 was congruent with the official ICTV taxonomy (Walker et al., 2020; Turner et al., 2021), as these groups clustered together in both the terminase and integrase trees, reflecting their shared membership in the *Peduoviridae* family.

The third group included a *Tlsvirus*-related subgroup. While this group was not represented in the integrase tree—likely due to its characterization as strictly lytic—its placement in the terminase tree aligns with previous reports. Specifically, its association with *Tlsvirus* F61 and *Tlsvirus* SP126 is consistent with prior studies identifying these phages pathogens of *S. infantis* in poultry farms (Parra et al., 2023).

Phylogenetic analysis based on individual TerL and TerS sequences (Supplementary Figures 3, 4) showed lower support for established relationships compared to the concatenated approach. This discrepancy likely reflects the inherent challenges in viral phylogenetics, given the high diversity, modular genomes, and variations between species depending on their host and environment (Rohwer and Edwards, 2002; Lima-Mendez et al., 2008; Mavrich and Hatfull, 2017; Dion et al., 2020). While the recommended approach for robust phage classification involves the use of complete genome sequences and protein cluster analysis (Lima-Mendez et al., 2008; Dion et al., 2020; Maffei et al., 2021), this method was not feasible for our study as several of the identified prophage sequences were incomplete. Consequently, we utilized the large and small subunits of the terminase, as these were the most prevalent markers across our dataset, an approach supported by Wangchuk et al. (2021). The integrase was used as a complementary marker only for reference genomes and sequences positive for this protein (Colavecchio et al., 2017). Although other markers such as capsid, integrases, and phage spanins have been proposed (Holmes, 2011; Kongari et al., 2018; Dion et al., 2020), they were not consistently identified in our prophage fragments. Therefore, given the fragmented nature of the data and the complexities of viral evolution, the use of concatenated terminase subunits represented a pragmatic approach to explore these relationships. Further research remains necessary to establish universally accepted consensus methods for viral phylogenetics.

## 5 Conclusion

Our study characterizes the prophage landscape of *Salmonella enterica* in Ecuadorian poultry environments, revealing a high degree of genomic fragmentation and diversity. The prevalence of specific prophages—notably *Peduovirus* pro483 in *S. infantis* and *ST104* in *S. Typhimurium*—suggests a potential role for these elements in serovar-specific adaptation.

Furthermore, the identification of virulence and superinfection exclusion genes, alongside the absence of SopE, suggests a specialized genomic profile in these lineages. The presence of fimbrial proteins in both the *Peduovirus* pro483 variants and the p-F219 megaplasmid may indicate a coordinated evolutionary strategy favoring colonization and niche persistence in the established *S. infantis* population of Ecuador. Although the fragmented nature of the prophage sequences necessitated a pragmatic phylogenetic approach using concatenated terminase subunits, this method effectively resolved the evolutionary relationships within our dataset, providing a foundation for future functional studies.

These findings highlight the potential of prophages as markers for strain evolution. While further research remains necessary—specifically by expanding this methodological framework to include a more diverse array of *Salmonella* genomes, including additional poultry-related serovars and their associated mobile genetic elements (MGEs), to explore less prevalent serovars in Ecuador—this *in silico* approach provides a foundation for understanding *Salmonella* genomic dynamics. Ultimately, this work informs the future development of targeted interventions in poultry production within the region.

## Data Availability

*Salmonella enterica* sequenced genomes are available under bioproject PRJEB37560 in the NCBI. The corresponding codification and serovar of every *S. enterica* genome and the quality control measures are presented in Supplementary Table 1. The custom database built for this study is listed with their corresponding accession numbers to NCBI in Supplementary Table 2. A list of every prophage detected for every *S. enterica* genome along with their corresponding codification and serovar. All alignments and other raw data used for the analysis in this study are deposited at: https://github.com/fran1722/Prophage-diversity-in-poultry-associated-Salmonella-enterica-from-Ecuador.git.

## Footnotes

1 Available at: https://github.com/fran1722/Prophage-diversity-in-poultry-associated-Salmonella-enterica-from-Ecuador.git.
