# Supplementary figures and images for: Prophage diversity in poultry-associated Salmonella enterica from Ecuador: a case study using an in-silico terminase-based approach
Source: Front Microbiol. 2026 Mar 12;17:1703134. doi: 10.3389/fmicb.2026.1703134 (PMC13017927; doi:10.3389/fmicb.2026.1703134)

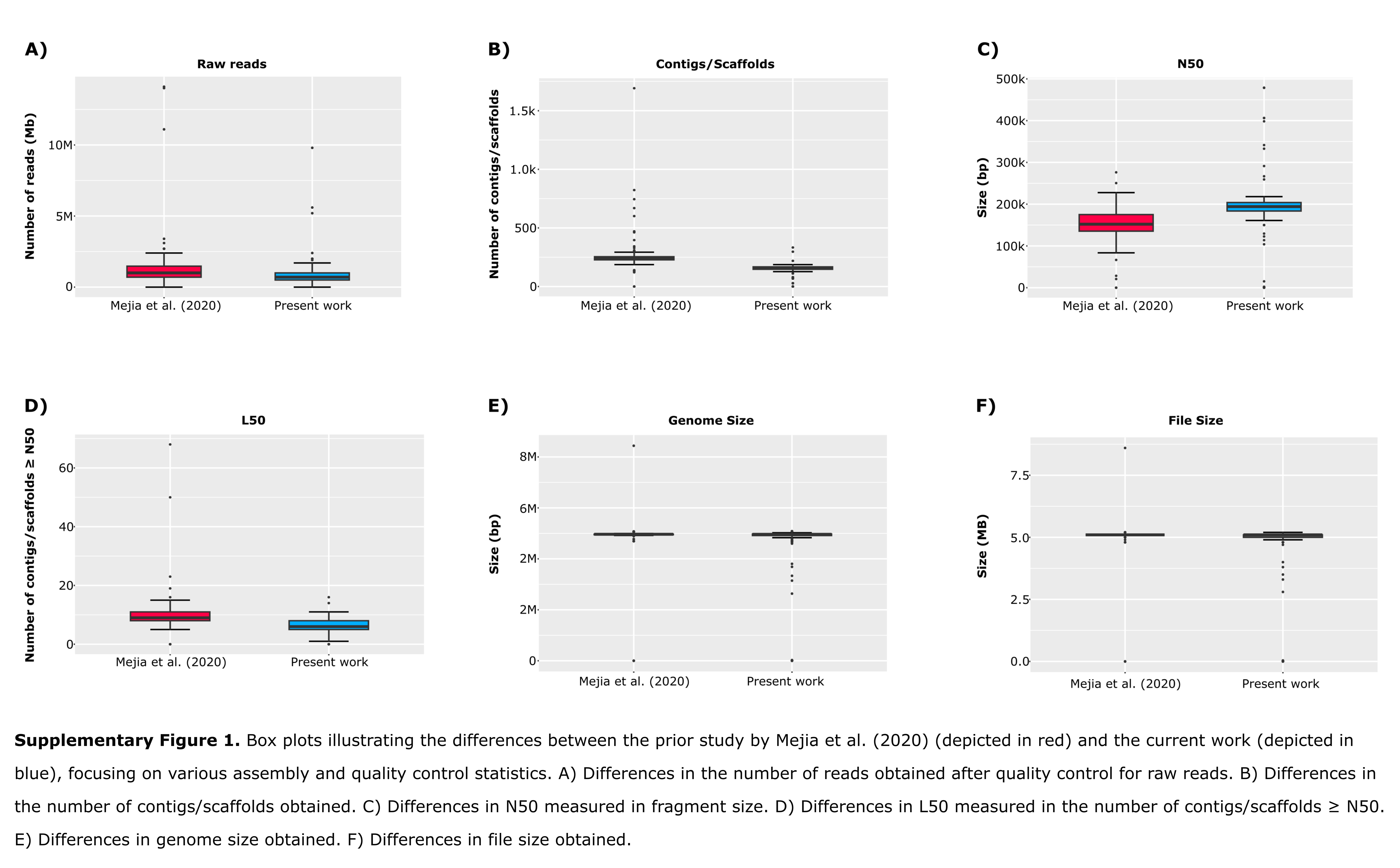

Supplement: Supplementary file 4 [file Image_1.JPEG]

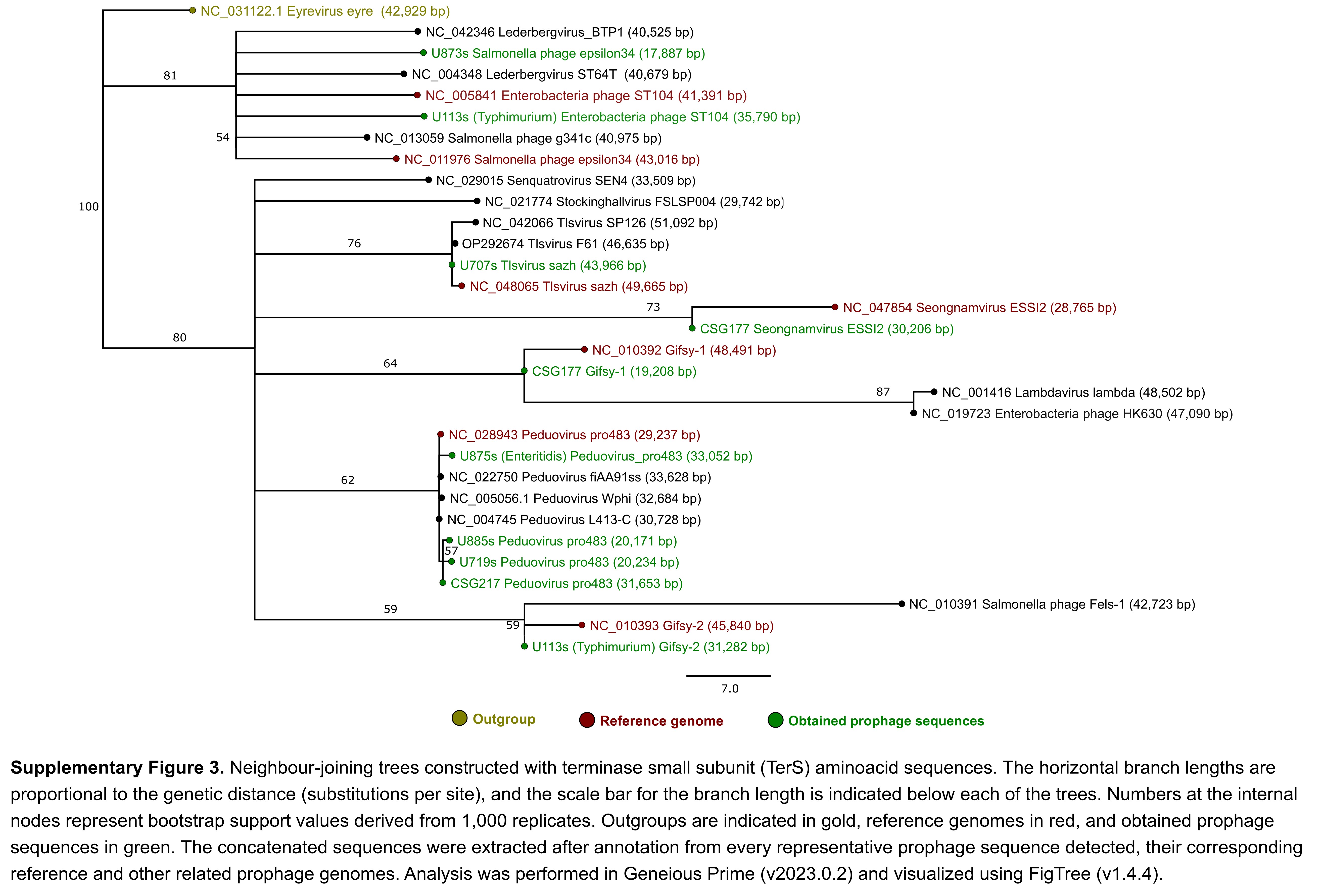

Supplement: Supplementary file 6 [file Image_3.JPEG]

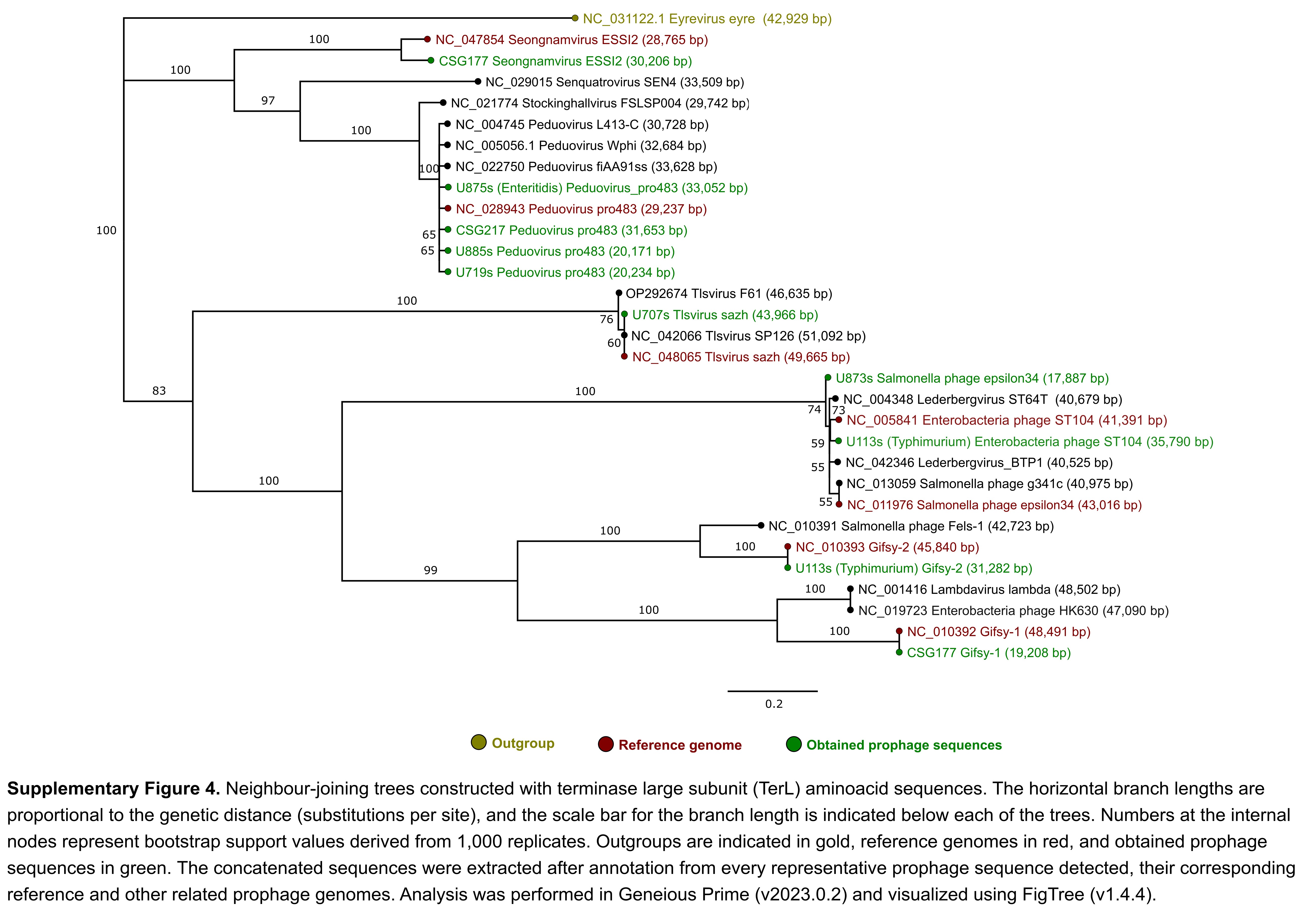

Supplement: Supplementary file 7 [file Image_4.JPEG]
